# Spectral Doppler, tissue Doppler, and speckle-tracking echocardiography for the evaluation of fetal cardiac function: an update

**DOI:** 10.1590/0100-3984.2020.0052

**Published:** 2021

**Authors:** Alberto Borges Peixoto, Nathalie Jeanne Bravo-Valenzuela, Luciane Alves Rocha, Edward Araujo Júnior

**Affiliations:** 1 Gynecology and Obstetrics Clinic, Hospital Universitário Mário Palmério - Universidade de Uberaba (Uniube), Uberaba, MG, Brazil.; 2 Department of Obstetrics and Gynecology, Universidade Federal do Triângulo Mineiro (UFTM), Uberaba, MG, Brazil.; 3 Division of Pediatrics (Pediatric Cardiology), Department of Internal Medicine, Universidade Federal do Rio de Janeiro (UFRJ), Rio de Janeiro, RJ, Brazil.; 4 Graduate Program in Health Sciences, Universidade Federal do Amazonas (UFAM), Manaus, AM, Brazil.; 5 Department of Obstetrics, Escola Paulista de Medicina da Universidade Federal de São Paulo (EPM-Unifesp), São Paulo, SP, Brazil.

**Keywords:** Fetal heart, Ultrasonography, Doppler, Echocardiography, Doppler, Echocardiography/methods, Coração fetal, Ultrassonografia Doppler, Ecocardiografia Doppler, Ecocardiografia/métodos

## Abstract

The functional assessment of the fetal heart has been incorporated into cardiac ultrasound screening as a routine procedure, encompassing fetuses with and without structural heart diseases. It has long been known that various cardiac and extracardiac conditions, such as fetal growth restriction, fetal tumors, twin-to-twin transfusion syndrome, fetal anemia, diaphragmatic hernia, arteriovenous fistula with high cardiac output, and congenital heart diseases (valvular regurgitation and primary myocardial disease), can alter hemodynamic status and fetal cardiac function. Several ultrasound and Doppler echocardiographic parameters of fetal cardiovascular disease have been shown to correlate with perinatal mortality. However, it is still difficult to identify the signs of fetal heart failure and to determine their relationship with prognosis. The aim of this study was to review the main two-dimensional Doppler ultrasound parameters that can be used in the evaluation of fetal cardiac function, with a focus on how to perform that evaluation and on its clinical applicability.

## Abstract (pt)

A avaliação funcional do coração fetal foi incorporada ao rastreamento ultrassonográfico como procedimento de rotina, englobando fetos com e sem cardiopatias estruturais. Sabe-se, tradicionalmente, que várias condições cardíacas e extracardíacas podem alterar o estado hemodinâmico e a função cardíaca fetal, como restrição do crescimento fetal, tumores fetais, síndrome de transfusão feto-fetal, anemia fetal, hérnia diafragmática congênita, fístula arteriovenosa com alto débito cardíaco e cardiopatias congênitas (insuficiência valvar, doença primária do miocárdio). Vários parâmetros ecocardiográficos da doença cardiovascular fetal obtidos por ultrassonografia e Doppler têm sido correlacionados com a mortalidade perinatal. No entanto, ainda é difícil determinar a presença de sinais de insuficiência cardíaca fetal e sua relação com o prognóstico. Este estudo teve como objetivo revisar os principais parâmetros bidimensionais da ultrassonografia Doppler que podem ser utilizados na avaliação da função cardíaca fetal, com foco em como realizar essa avaliação e sua aplicabilidade clínica.

## INTRODUCTION

### Basics of cardiac function

The heart is composed of four chambers: the right atrium, right ventricle (RV), left atrium, and left ventricle (LV). The atria receive venous blood, whereas the ventricles eject blood into the systemic circulation. Once the intraventricular pressure falls below the atrial pressure, the atrioventricular (AV) valves open and blood flows into the ventricles as a result of depolarization and atrial contraction. The terms active filling and passive filling are used in reference to the macroscopic appearance, given that both processes are molecularly active ^(1)^.

As the heart rate increases, the atria increase their contribution to cardiac output by shortening the passive ventricular filling time. In situations in which the heart rate increases, to counter the reduction in the passive filling time of the ventricles, the sympathetic nervous system, which is responsible for the positive inotropic effect, also increases the ventricular relaxation time by decreasing the duration of the action potential of myocardial muscle fibers^(1)^.

As the ventricles undergo depolarization and contraction, there is a progressive increase in intraventricular pressure and closure of the AV valves, after which isovolumetric contraction begins. The isovolumetric contraction time (ICT) is the time between the closure of AV valves and the opening of aortic and pulmonary valves, which results in blood being pumped into the pulmonary and systemic circulation. The semilunar valves open when the intraventricular pressure exceeds the pressure within those valves. As the ventricular contractility decreases, the intraventricular pressure decreases and the semilunar valves close. The time between the closure of the semilunar valves and the opening of the AV valves is known as isovolumetric relaxation time (IRT). During ventricular systole, the atria fill continuously, causing the atrial pressure to increase until it exceeds the ventricular pressure, the AV valve to open, and a new cycle to begin^(1)^. From a cardiac function point of view, systolic changes alter the ICT, whereas diastolic changes affect the IRT^(2)^.

Systolic volume and the amount of blood pumped by the heart during a single heartbeat depend mainly on three factors: preload, afterload, and contractility. Preload is the ventricular end-diastolic pressure. It is the main determinant of ventricular volume and, consequently, of the distention capacity of the ventricular muscle fiber. According to the Frank-Starling law, a normal heart increases its contractility when the distention of myocardial muscle fibers increases. Therefore, an increase in ventricular end-diastolic volume causes an increase in systolic volume. Afterload is the pressure that the heart muscle fibers must overcome to allow the pumping of blood. Therefore, an increase in afterload decreases ventricular contractility, thus reducing systolic volume. Systemic blood pressure is the main contributor to increased afterload. Finally, contractility is the ability to shorten muscle fiber and depends on the action of the sympathetic nervous system. The release of norepinephrine increases the contractility of the heart and, consequently, systolic volume^(1)^.

Various parameters have been developed for the assessment of cardiac function. They were initially developed for use in adults and later adapted for the evaluation of fetal hearts. Some parameters use Doppler flow mapping, whereas others use cardiac biometrics, cardiac cycle time, or a combination of the two. The main parameters are as follows^(1)^: stroke volume, cardiac output, ejection fraction, relaxation fraction, myocardial contractility, and myocardial performance index (MPI). Table 1 shows the formulas for determining the parameters employed in the assessment of fetal cardiac function.

**Table 1:** Formulas for the calculation of the parameters employed in the assessment of fetal cardiac function.

Parameter	Formula
Stroke volume	Valvediameter×totalvelocitytime
Cardiac output	Strokevolume×heartrate
Ejection fraction	Strokevolume/end−diastolicvolume
Shortening fraction	$\left(\mathrm{VEDd}-\mathrm{VESd}\right)/\mathrm{VEDd}$
MPI	$\left(\mathrm{IVCT}+\mathrm{IVRT}\right)/\mathrm{ET}$

VEDd, ventricular end-diastolic diameter; VESd, ventricular end-systolic diam­eter.

Mielke et al.^(3)^ evaluated stroke volume and cardiac output in 222 fetuses from 13 weeks of gestation to term. The authors demonstrated that stroke volume and cardiac output increased exponentially with advancing gestational age. The median cardiac output in both ventricles ranged from 40 mL/min at 15 weeks to 1,470 mL/min at 40 weeks gestation. The median fetal weight-corrected cardiac output was 425 mL × min^−1^ × kg^−1^, and the median cardiac output ratio between the RV and LV was approximately 1.4, remaining stable during pregnancy, the RV being dominant.

### Main techniques for the assessment of fetal cardiac function

In the past, fetal cardiac function was assessed by measuring the ejection fraction and ventricular shortening fraction through the use of M-mode and two-dimensional (2D) ultrasound. However, the use of those techniques before birth had some limitations because of the small dimensions of the fetal heart, movement artifacts, inappropriate fetal position, and the geometric complexity of the fetal RV^(4)^. Due to the greater technical difficulty in the use of conventional markers of cardiac function, there has been increasing interest in the study of alternative methods, such as determination of the AV blood flow (early/atrial [E/A] ratio) and the MPI, as well as spectral tissue Doppler, all of which allow evaluation of the contractility and degree of deformity of the fetal heart musculature.

### E/A ratio

**Figure 1:**
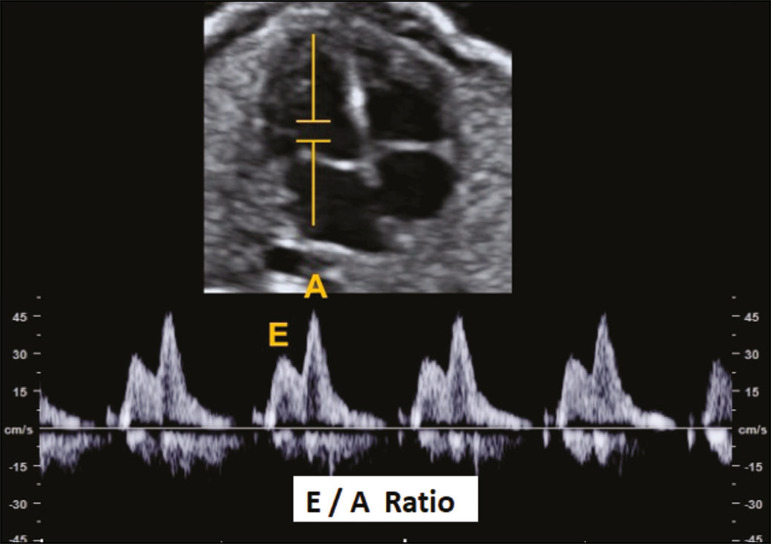
Spectral Doppler imaging showing the E and A waves through the fetal heart mitral valve.

The E/A ratio represents the relationship between the early and late peak flow velocity through the AV (mitral and tricuspid) valves during diastole. Determining the E/A ratio with spectral Doppler allows evaluation of the ventricular diastolic component of the cardiac cycle. To calculate the E/A ratio, it is necessary to place the spectral Doppler sample volume just below the AV valves. As shown in Figure 1, a biphasic wave will be observed^(4)^; its first component is the E wave, which represents the passive filling phase, whereas its second component (the A wave) represents the active filling phase.

The biphasic form of blood flow through the AV valves can be identified as of 9 weeks of gestation^(5)^. In healthy fetuses, the peak velocity of the A wave is generally higher than is that of E wave. As gestational age advances, the E and A wave velocities increase, the increase in E wave velocity being more pronounced because of the greater capacity of the fetal heart for compliance and relaxation^(6,7)^. This pattern of blood flow through the AV valves manifests as progressive elevation of the E/A ratio through pregnancy. A reduction in the E/A ratio indicates that the ventricular filling process depends more on the atrial contraction than on the negative pressure exerted during the relaxation phase and may therefore also indicate impaired diastolic ventricular function^(8)^.

#### Technical aspects

The E/A ratio should be evaluated in the four-chamber view of the heart, in the absence of fetal body and respiratory movements. The sample volume should be 2-3 mm and should be positioned distal to the AV valves, preferably with an insonation angle < 20°. The pulse repetition frequency must be adjusted so that the velocity wave occupies at least 75% of the scale and that 3-5 symmetrical waves are visible^(6)^.

### MPI

The MPI is calculated, by spectral Doppler, with the following formula:

$$\mathrm{MPI}=\left(\mathrm{ICT}+\mathrm{IRT}\right)/\mathrm{ET}$$

where *ET* is the ejection time. The MPI is capable of assessing overall cardiac function because it comprises the systolic and diastolic components of the cardiac cycle and can be used in order to assess the two ventricles separately. The MPI values for the fetal LV remain relatively stable and vary slightly during gestation, the mean being 0.36, with a standard deviation of 0.28-0.44^(9)^. The ICT represents the time between ventricular contraction and the opening of the aortic and pulmonary valves. During the ICT, there is increased intraventricular pressure without changes in the volume of the RV or LV. The IRT begins with diastole after closure of the aortic and pulmonary valves. At that moment, there is ventricular isovolumetric relaxation but no entry or exit of blood; therefore, the intraventricular pressure gradually declines. The ET starts when the intraventricular pressure is sufficient to promote the opening of the aortic and pulmonary valves, causing myocardial deformation and blood to gain systemic circulation^(9)^.

Myocardial dysfunction increases the MPI values through prolongation of the IRT and reduction of the ET^(10)^. The IRT is the main parameter that changes during the early phase of myocardial dysfunction, the change being due to the suppression of calcium reuptake by cardiomyocytes, which prolongs the time required for complete myocardial relaxation^(11,12)^.

#### Applicability in the evaluation of fetal cardiac function

Tsutsumi et al.^(13)^ were the first to use MPI to assess overall fetal cardiac function by analyzing two waves during the same cardiac cycle. One subsequent study showed that there is great variability when this measurement technique is employed^(14)^. Friedman et al.^(10)^ proposed a method for assessing MPI by positioning the spectral Doppler sample volume near the mitral influx and aortic outlet. Thus, it would be possible to evaluate the isovolumetric and ejection periods simultaneously during the same cardiac cycle.

**Figure 2:**
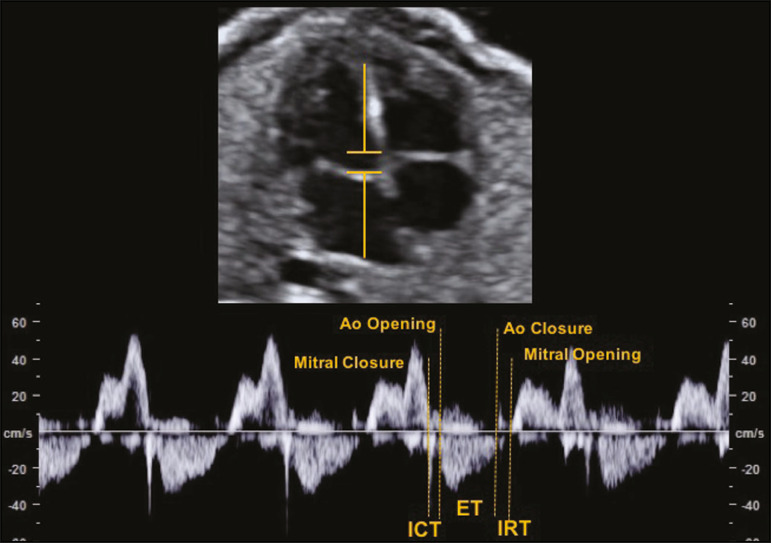
MPI measured by aperture clicks in the Doppler velocity spectrum.

**Figure 3:**
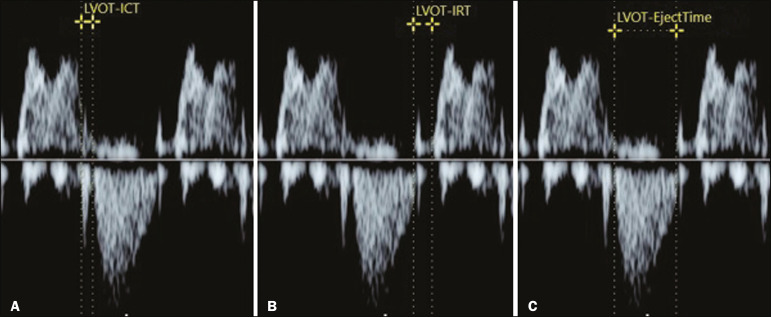
Reference speckle (acoustic point) for measuring the modified MPI using the opening and closing clicks of the fetal heart mitral and aortic valves. A: ICT measured from the beginning of the mitral valve closure click to the beginning of the aortic valve opening click. B: IRT measured from the beginning of the aortic valve closure click and beginning of the mitral valve opening click. C: ET measured from the beginning of the aortic valve (opening click) to the beginning of the aortic valve (closing click).

The opening and closing times of the valve leaflets produce echoes, or "clicks", visible as vertical stripes on the spectral Doppler velocity profile. Opening and closing of the valve leaflets produce the "original" clicks, located in the same direction as the blood flow (opening clicks) or in the opposite direction (closing clicks). Small echoes know as "reflected" clicks can be identified in the opposite direction from the original clicks. Original and reflected clicks share the same peak point (Figure 2). Raboissonn et al.^(15)^ used mitral and aortic valve opening clicks as references to improve the estimation of the time intervals for MPI calculation. Hernandez-Andrade et al.^(16)^ described the use of a modified MPI, which was used as a reference to identify different time points: the beginning of the opening clicks and the beginning of the closing clicks (Figure 3). That technique improved the reproducibility of MPI by reducing interobserver and intraobserver variability.

The MPI has been used in order to assess fetal cardiac dysfunction in a number of conditions, including fetal growth restriction^(17)^, pregestational diabetes mellitus^(18)^, twin-to-twin transfusion syndrome^(19)^, pre-eclampsia^(20)^, and congenital heart diseases^(21)^. It is a reliable parameter for identifying early cardiac dysfunction and early changes related to fetal cardiac adaptation to various perinatal insults. Increased MPI is rarely transient, and the MPI remains high in complicated cases^(8)^.

#### Technical aspects

During evaluation of the MPI, manual positioning of calipers on the Doppler velocity wave is used in order to evaluate the time intervals between the various clicks. Minor variations in caliper position can lead to millisecond variations in time intervals and, consequently, significant changes in the MPI values^(22)^. In the literature, there is no consensus on the exact caliper positioning at the moment of MPI measurement. That inconsistency in the methodology contributes to the wide variation between normal values and consequent difficulty in comparing the results across studies^(14)^. Although the protocol for the most widely used technique for measuring the MPI states that the calipers should be positioned at the beginning of the opening click and at the beginning of the closing click^(16)^, there is still no consensus on the literature. In one study, the calipers were positioned to span the space between the end of one click and the beginning of the next, the authors arguing that this form of measurement would be the most physiological, given that it does not include the period of valve movement in the calculation of ICT and IRT^(23)^. In another study, the calipers were positioned at the peak of the click^(24)^. The use of peak clicks improved the reproducibility of MPI measurement by establishing a clearer reference point for measuring ICT, IRT, and ET, thus avoiding variations due to differences in the width of the valve clicks^(24)^.

Another technical detail that also contributes to the variation in the mean MPI values for the LV is the differences between the settings and ultrasound device brands^(25)^. Table 2 shows the comparison of the different MPIs obtained in studies using the Siemens Antares system (Siemens Medical Solutions, Mountain View, CA, USA), the GE Voluson 730 Expert system (GE Healthcare, Milwaukee, WI, USA), or both, as well as using a variety of settings. To establish the best setting to ensure high repeatability of MPI values with the Siemens Antares and GE Voluson 730 Expert systems, Lobmaier et al.^(25)^ conducted a prospective cohort study of 62 fetuses between 28 and 36 weeks of gestation in women with low-risk pregnancies. The authors showed that sweep speed and the filter setting were the parameters that had the greatest influence on the MPI value. Reduced scanning speed resulted in less reproducibility, because it reduced the accuracy of the determination of the time intervals (ICT, IRT, and ET). Lowering the filter worsened reproducibility of the MPI, which improved when the filter was raised. When the filter was too low, artifacts appeared near the baseline, making it difficult to identify the beginning of the valve opening and closing clicks. In contrast, raising the filter allowed better click identification and eliminated the blurred area near the baseline. The authors found that modifying the gain had little effect on MPI variability. In fact, they showed that the gain adjustment, which largely depended on maternal body mass index and fetal position, could be individually adjusted to make the sonographer feel more confident in identifying valve clicks. The authors also stated that the best device settings to ensure the highest reproducibility seemed to be a sweep speed of 8 cm/s, a gain of 60 dB, and a 281-Hz filter for the Siemens Antares system, compared with a sweep speed of 5 cm/s, a gain of −10 dB, and a 210-Hz filter for the GE Voluson 730 Expert system.

**Table 2:** Different values of MPI between settings and brand of ultrasound devices used in different studies.

Study	MPI Mean (SD)	Angle of insonation (º)	WMF (Hz)	Sample volume (mm)	Sweep velocity (cm/s)	Doppler gain	Ultrasound device brand
Hernandez-Andrade et al.**^(9)^**	0.37 (0.03)	< 30	70	3	15	Minimum	Siemens Antares, GE Voluson 730 Expert
Van Mieghem et al.**^(23)^**	0.34 (0.05)	< 15	> 120	No data	10	No data	GE Voluson 730 Expert
Meriki et al.**^(22)^**	0.39 (0.55)	< 15	300	3	15	Minimum	GE Voluson E8
Cruz-Martínez et al.**^(26)^**	0.41	No data	Low	2-4	Maximum	Minimum	Siemens Antares, GE Voluson 730 Expert
Lobmaier et al.**^(25)^**	0.44 (0.05)	< 15	281	4	15	60	Siemens Antares
Lobmaier et al.**^(25)^**	0.44 (0.05)	< 15	210	4	15	-10	GE Voluson 730 Expert
Peixoto et al.**^(27)^**	0.46 (0.01)	< 20	210	4	15	-10	GE Voluson E8, GE Voluson E6

SD, standard deviation; WMF, wall motion filter.

### Tissue Doppler

Tissue Doppler is a reproducible technique that allows analysis of myocardial motility by assessing the peak velocities of contraction and expansion of a myocardial segment by positioning the spectral Doppler sample volume in the mitral and tricuspid annuli^(1)^. It enables simultaneous evaluation of the systolic and diastolic components of the cardiac cycle and provides a good approximation of ventricular contractility by analyzing the motility of the AV valve annulus^(28)^.

**Figure 4:**
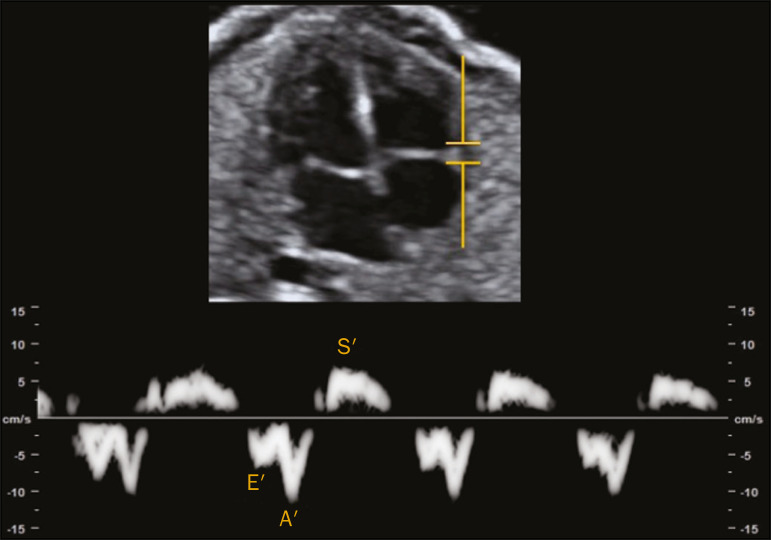
Peak velocity obtained by spectral tissue Doppler in the right annulus of the fetal heart.

**Figure 5:**
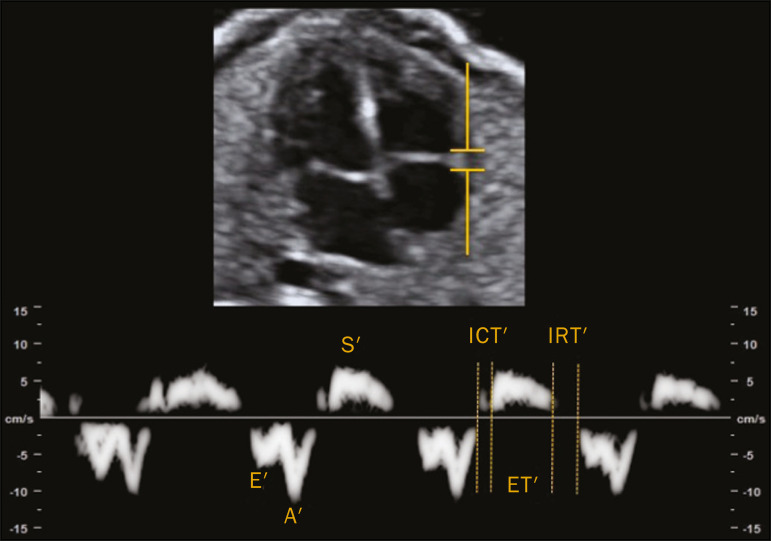
Measurement of peak velocities and RV tissue MPI′ by the assessment of tissue ICT′, tissue IRT′, and tissue ET′.

Assessment of the longitudinal motility of the AV valve annulus provides three velocity waves (Figure 4): the S′ wave, which represents the annular peak velocity during ventricular systole; the E′ wave, which represents the annular peak velocity during passive filling; and the A′ wave, which represents the annular peak velocity during active filling. Harada et al.^(29)^ were the first to establish the reference values for fetal cardiac function, as well as to describe changes in RV myocardial velocity waves, LV septum, and interventricular septum in the fetal heart. Under normal conditions, the A′ wave has a peak velocity greater than that of the E′ wave. As gestational age advances, there is a decrease in the E′/A′ ratio due to changes in ventricular compliance that can be identified from the AV valve velocity pattern. In addition to the annular peak velocities, it is possible to calculate the tissue MPI′ through spectral tissue Doppler by analyzing the time periods of the velocity spectrum (Figure 5): ICT′, ET′, and IRT′. The MPI′ can be calculated by using the following formula:

MPI'=ICT'+IRT'/ET'

#### Technical aspects

To perform spectral tissue Doppler and obtain the speed spectrum, it is necessary to magnify the image of the four-chamber view of the fetal heart (apical or basal view). In addition, position the Doppler sample volume (2-4 mm) should be positioned at the basal segment of the RV or LV (tricuspid or mitral annulus). The insonation angle between the ultrasound beam and ventricular wall or interventricular septum should be < 30°, and no correction angle should be used. The filter and gain should be set low to avoid high-frequency signals. It is also necessary to obtain at least three speed waves in order to perform a proper analysis of the results^(30)^.

The limitations for performing spectral tissue Doppler in fetal cardiology include improper fetal positioning, inappropriate angle of insonation, small size of the fetus, high fetal heart rate, and the low resolution capacity of some ultrasound devices. Despite those limitations, several recent studies have shown that spectral tissue Doppler is a reliable, reproducible method^(29-31)^. The main limitation of spectral tissue Doppler is that it evaluates the velocity of only a small portion of the myocardium and does not analyze the capacity or rate of myocardial deformation. The advantage of spectral tissue Doppler is that it allows a more accurate assessment of cardiac contractility by analyzing a segment of the myocardium rather than changes in cardiac chamber dimensions^(32)^.

### Speckle tracking

One echocardiographic imaging technique is 2D speckle tracking (2DST), which analyzes the motion of tissues in the heart on the basis of the speckle pattern in the myocardium. That speckle pattern is a mixture of interference patterns and natural acoustic reflections. It can be described as tracking a speckle (acoustic point) in a 2D image^(33,34)^. In the grayscale 2D image, that acoustic point changes position as myocardial deformity occurs during the cardiac cycle.

In 2DST, the acoustic point is tracked through the acquisition of 2D cine images of the cardiac cycle, which are analyzed frame by frame throughout the cycle^(35)^. The movement of the point creates vectors with different orientations. This technique is independent of the insonation angle^(34,35)^, because, unlike tissue Doppler, it does not require a Doppler signal in order to study the displacement of the acoustic points^(34)^.

#### Technical aspects

For proper acquisition of a 2D cine images of the cardiac cycle film, it a frame rate of approximately 40-80 fps should be used. Using lower frame rates can result in a loss of acoustic point tracking quality. Caution must be taken when maximizing cardiac imaging using the depth and zoom tools, because the myocardial borders may be lost.

Displacement of the acoustic point is called myocardial deformity or strain and is expressed as a percentage. It may be negative or positive depending on whether the myocardial fibers are shortening or lengthening, respectively. The speed with which this displacement occurs can also be measured (in cm/s). The movement of each acoustic point can be plotted on a curve as a function of time, known as strain rate, expressed in 1/s or s^−1^. The path taken by the point can be analyzed in orthogonal and tangential planes^(34)^. The first orthogonal plane described here evaluates the longitudinal strain rate. In the longitudinal plane, there is shortening of the cardiac cavities and deformity of the myocardium in the basal-apical direction. The cavity is smaller during systole than during diastole, so the deformation in the longitudinal plane is negative^(34,35)^. In the radial plane, we evaluated the wall thickness on the short axis of the ventricles, which is called radial strain. Because the systolic thickness is greater than the diastolic thickness, the deformation in the radial plane is positive^(34,36)^. The circumferential plane is also assessed on the short axis of the ventricles. The circumferential dimension of the cavities is known as circumferential strain. The circumference is smaller in systole than in diastole, so the deformation in the circumferential plane is also negative^(34,36)^. In tangential planes, the displacement of acoustic points is evaluated in the epicardium and endocardium, in order to demonstrate the slip between the layers of the ventricle, although that technique is still in the validation phase^(34,35)^.

#### Applicability in the evaluation of fetal cardiac function

In the literature about the evaluation of fetal cardiac function, 2DST figures prominently^(37)^. The method has great potential for the evaluation of diseases that impair myocardial contractility and can detect early changes in cardiac function prior to the age at which cardiovascular diseases would typically be diagnosed. It is a technique that may improve the understanding of what occurs in myocardial fibers during the cardiac cycle in fetuses. Multiple studies have been conducted to establish normal values that serve as a starting point to analyze cardiac function using this technique and thus allow such diseases to be identified in the prenatal period^(38)^.

To date, there are limited data on the strain and strain rate in healthy fetuses, as well as on the evolution of those measures throughout pregnancy. Using velocity vector imaging, Barker et al.^(39)^ assessed global longitudinal cardiac strain and strain rate in 33 healthy fetuses and 15 fetuses with heart disease. The authors found that segmental measurements were not significantly different from global measurements; therefore, global measurements may be a useful tool to evaluate fetal cardiac function. Peng et al.^(40)^ evaluated 151 normal fetuses and found that strain and strain rate can be measured easily and in a reproducible manner. However, they reported that the values for RV and LV global longitudinal strain decreased throughout gestation. It is of note that, throughout pregnancy, strain values are higher in the LV than in the RV^(38)^. In contrast, strain rates seem to remain stable and similar in both ventricles throughout gestation^(38)^.

## CONCLUSION

A variety of cardiac ultrasound/echocardiographic parameters can be used in order to identify fetal heart failure and predict the risk of adverse perinatal outcomes. The shortening fraction enables the evaluation of ventricular global systolic function (radial contractility). However, the shortening fraction is altered in the late stages of myocardial dysfunction and its determination is impaired by technical limitations in myocardial asymmetry, such as fetal growth restriction and fetuses in women with pregestational diabetes. The MPI is used in order to assess systolic and diastolic cardiac function and can be calculated by analyzing the time periods in the cardiac cycle, as determined with spectral or tissue Doppler. Despite the clinical applicability of the MPI, there is variability among reference values in the literature. Consequently, it is important to minimize variations among ultrasound systems for the measurement of MPI by using the proper presets established by the sonographer and echocardiographer for each system. Spectral and tissue Doppler imaging of AV valve flow can be used for the assessment of diastolic cardiac function, in which the E/A and E′/A′ ratios reflect the ventricular compliance. In such assessments, tissue Doppler is more accurate than is spectral Doppler and enables the segmental analysis of diastolic myocardial function. Given that the diastolic ventricular inflow can be transmitted to the atrium, Doppler imaging of the pulmonary veins and inferior vena cava can be also used in this analysis. Other advanced techniques, such as 2DST and strain imaging, may be used in the assessment of the degree of deformity of the myocardium. Therefore, the evaluation of fetal cardiac function should encompass a variety of Doppler ultrasound/echocardiographic parameters that can be selected on the basis of specific fetal conditions.
